# Of Rats and Men: Poussin’s Plague at Ashdod

**DOI:** 10.3201/eid2401.AC2401

**Published:** 2018-01

**Authors:** Victor Asensi, Joshua Fierer

**Affiliations:** Oviedo University School of Medicine, Oviedo, Spain (V. Asensi);; VA San Diego Healthcare System, San Diego, California, USA (J. Fierer);; University of California San Diego School of Medicine, San Diego (J. Fierer)

**Keywords:** art science connection, emerging infectious diseases, art and medicine, about the cover, Nicholas Poussin, Of rats and men: Poussin’s plague at Ashdod, The Plague at Ashdod, Book of Samuel, Alexandre Yersin, Kitasato Shibasaburo, plague, bubonic plague, Yersinia pestis, bacteria, Rattus, rats, fleas, pathogenesis, vector-borne infections, zoonoses

**Figure:**
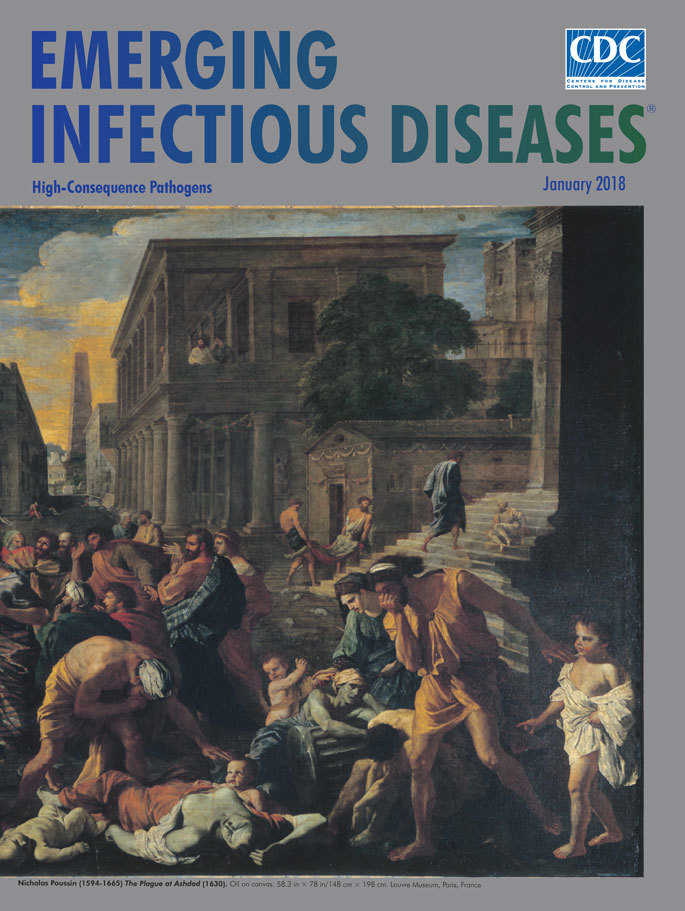
**Nicolas Poussin (1594−1665). The Plague at Ashdod, 1630.** Oil on canvas. 58.3 in × 78 in/148 cm × 198 cm. Louvre Museum, Paris, France

Nicolas Poussin (1594–1665) was a brilliant French Baroque painter whose art was inspired by biblical and mythological scenes. Poussin depicts the *Plague at Ashdod* (1630) (Louvre Museum, Paris, France) in one of his best works, inspired by an episode from chapter 5 of the Book of Samuel. On this large canvas, rats run through buildings and among dead and dying bodies. The Book of Samuel, written during 630–540 bce, recounts the capture of the Ark of the Covenant by the Philistines, who moved it to the city of Ashdod. “Soon after receiving the Ark rats appeared in the land and death and destruction spread throughout Ashdod. The Philistines, young and old, were struck by an outbreak of tumors in the groin and died.” The Philistines sent the Ark back to Israel with a guilt offering of “five gold tumors and five gold rats,” models of the pestilences destroying the country.

This biblical text has been linked to bubonic plague by some, but not all, authors because black rats from the Far East did not reach the Near East until the 1st century bce. However, fossilized remains of *Xenopsylla cheopis* fleas and the *R.* *rattus* black rats have been found in the Egyptian Nile Valley, dating their arrival in the Middle East to 1350 bce. The Jewish–Roman historian Flavius Josephus (37−100 ace) attributed the epidemic to bacillary dysenter, which can lead to hemorrhoids, his translation of the Hebrew word “opalim.” However, Josephus’ translation of the Hebrew word has been questioned. The original Hebrew text of the Book of Samuel uses two words to describe the plague’s pathology, namely *techorim* (tumor) and *ophel* (boil), both appropriate for bubonic plague.

The King James version of the Bible translates both words as “emerods” (hemorrhoids), and the New International version of the Bible translates both as “tumors.” The Septuagint, a Hebrew-to-Greek translation of the Torah made in the 3rd century in Egypt by 72 Hebrew scholars, and Saint Jerome’s translation of this Greek text into Latin, both expand on the original Hebrew by stating that the tumors were in the groin (*bubo* is derived from the Greek word for groin). The Septuagint translation by Hebrew scholars seems more reliable than the translation to Latin by Josephus.

It is startling that in 1630 Poussin implicated rats in the pathogenesis of the bubonic plague, a fact disregarded until the end of the 19th century. Poussin lived through the Thirty Years’ War in France and Italy and might have seen cases of plague.

It was not until 1894 that Alexandre Yersin and Kitasato Shibasaburo, independently in Hong Kong isolated the bacterium responsible for the Third Bubonic Plague Pandemic. Yersin named it *Pasteurella pestis* after the Pasteur Institute, but in 1967 it was moved to a new genus, and renamed *Yersinia pestis* in honor of Yersin. Yersin also noted that rats were affected by plague during human epidemics. Plague was regarded in Asia as a disease of rats. Thus, when large numbers of rats were found dead, plague outbreaks soon followed.
